# Ion channels as molecular targets of glioblastoma electrotherapy

**DOI:** 10.3389/fncel.2023.1133984

**Published:** 2023-03-17

**Authors:** Tayeb Abed, Katrin Ganser, Franziska Eckert, Nicolai Stransky, Stephan M. Huber

**Affiliations:** ^1^Department of Radiation Oncology, University of Tübingen, Tübingen, Germany; ^2^Department of Radiation Oncology, Medical University Vienna, Vienna, Austria; ^3^Department of Pharmacology, Toxicology and Clinical Pharmacy, Institute of Pharmacy, University of Tübingen, Tübingen, Germany

**Keywords:** alternating electric fields, EMF, electrolocation, ampullae of Lorenzini, tuberous organs, voltage sensor

## Abstract

Therapies with weak, non-ionizing electromagnetic fields comprise FDA-approved treatments such as Tumor Treating Fields (TTFields) that are used for adjuvant therapy of glioblastoma. *In vitro* data and animal models suggest a variety of biological TTFields effects. In particular, effects ranging from direct tumoricidal, radio- or chemotherapy-sensitizing, metastatic spread-inhibiting, up to immunostimulation have been described. Diverse underlying molecular mechanisms, such as dielectrophoresis of cellular compounds during cytokinesis, disturbing the formation of the spindle apparatus during mitosis, and perforating the plasma membrane have been proposed. Little attention, however, has been paid to molecular structures that are predestinated to percept electromagnetic fields—the voltage sensors of voltage-gated ion channels. The present review article briefly summarizes the mode of action of voltage sensing by ion channels. Moreover, it introduces into the perception of ultra-weak electric fields by specific organs of fishes with voltage-gated ion channels as key functional units therein. Finally, this article provides an overview of the published data on modulation of ion channel function by diverse external electromagnetic field protocols. Combined, these data strongly point to a function of voltage-gated ion channels as transducers between electricity and biology and, hence, to voltage-gated ion channels as primary targets of electrotherapy.

## 1. Introduction

Tumor cells express an ion channel toolkit that differs from that of the non-transformed parental cells. Few ion channel types are upregulated by several tumor entities of different origin (carcinomas, leukemias, gliomas). These so-called “oncochannels” have been ascribed specific functions in tumor biology. For instance, glioblastoma, a primary brain tumor with poor patient prognosis, over-expresses TRPM8 (member 8 of the melastatin sub-family of transient receptor potential) unselective cation channels, intermediate conductance IK_Ca_ (*KCNN4*) and high conductance BK_Ca_ (*KCNMA1*) K^+^ channels. These channels reportedly contribute to glioblastoma stem cell properties, program and execute cell migration and brain invasion, regulate cell cycle, or confer therapy resistance (for review see Huber, 2013; Roth and Huber, 2022). Unexpectedly, ionizing radiation in a clinical relevant dose may induce hypermigration of human glioblastoma cells *in vitro* (Steinle et al., 2011) and brain invasion in an orthotopic glioma xenograft mouse model (Edalat et al., 2016). Notably, radiation-induced BK_Ca_ K^+^ channel activation has been identified as a key event herein (Steinle et al., 2011; Edalat et al., 2016). This example illustrates that modulation of oncochannel function by direct therapeutical targeting or as an off-target effect might exert beyond intended but also unintended, detrimental effects, due to the versatile functions of oncochannels (Huber, 2013). In this context, some electrotherapies applying weak external electromagnetic fields (EMF) have been proposed to affect ion channel function (e.g., Li et al., 2020a). The present review article, therefore, aims to summarize our current knowledge on ion channel modulation by EMF with a particular focus on tumor treating fields (TTFields), an adjuvant electrotherapy in glioblastoma.

*In vitro* (e.g., Rozek et al., 1987; García-Sancho et al., 1994; Morgado-Valle et al., 1998; Aldinucci et al., 2000; Djamgoz et al., 2001; Verdugo-Díiaz et al., 2002; Rosen, 2003; Grassi et al., 2004; Giladi et al., 2014a, 2017; Bertagna et al., 2022; Toda et al., 2022) and preclinical *in vivo* studies (e.g., Kirson et al., 2007; 2009a; Giladi et al., 2014a, b; Buckner et al., 2015; Castellví et al., 2015; Li et al., 2016; Voloshin et al., 2016, Jo et al., 2018; Kim et al., 2018; Voloshin et al., 2020a; Barati et al., 2021; Wu et al., 2021; Davidi et al., 2022; Huegel et al., 2022), as well as randomized controlled clinical trials (e.g., Mooney, 1990; Linovitz et al., 2002; Foley et al., 2008; Stupp et al., 2017), have accumulated some evidence for a responsiveness of physiological processes to weak external EMF. As a consequence, the US Food and Drug Administration (FDA) has approved electrotherapies in the low frequency to radiofrequency band and the non-hyperthermia-inducing, non-electroporating, non-ionizing energy spectrum. Among those are TTFields, repetitive transcranial magnetic stimulation (rTMS), deep brain stimulation (DBS), or pulsed electromagnetic fields (PEMFs). The latter are applied to improve the healing of bone fractures. PEMF and further EMF therapies used for orthopedic applications have been hypothesized to mimic the endogenous environmental electromagnetic fields occurring during mechanical stress of joints/bones. By doing so, they might exert pro-anabolic effects resulting in an increase of the structural integrity of bone and cartilage extracellular matrix (Cadossi et al., 2020). A Cochrane review of electromagnetic stimulation, in contrast, found only inconclusive evidence regarding its efficacy on bone fracture healing (Griffin et al., 2011). Similarly, early results of DBS in patients with treatment-resistant depression were highly promising (Mayberg et al., 2005), whereas a subsequent large sham-controlled randomized trial, could not delineate significant antidepressant effects of DBS (Holtzheimer et al., 2017). In a renewed attempt to utilize DBS for treating depressive symptoms, individualizing types of electrical stimulation is increasingly tested in new trials (Drew, 2022; Sheth et al., 2022).

Due to their limited (or even un-proven) stand-alone effects, weak EMF electrotherapies could not replace conventional therapies but have been implemented in the clinical routine as modalities adjuvant or complementary to the conventional treatments (for review see Mattsson and Simkó, 2019). For the different established electrotherapies, a huge spectrum of biological responses has been reported in preclinical studies. This is not surprising considering the diversity in frequency (ranging from static magnetic or electric fields, i.e., 0 Hz, to, e.g., 100 kHz of TTFields), pulse form (sinus, square, etc.), application (pulsed or continuous), or intensity of the individual EMF treatments. Since these therapies often operate with very low intensities, the molecular mode of action is mostly ill-defined and the physics underlying the electrobiological interaction difficult to deduce (for review see Mattsson and Simkó, 2019). As an example, weak magnetic fields with low frequency have been reported in several *in vitro* studies to exert maximal biological effects when their frequencies match the “cyclotron resonance” of certain ions, such as Ca^2+^, Mg^2+^, or K^+^ (for review see Liboff, 2018). At the cyclotron resonance frequency, the ratio of the applied alternating magnetic field frequency to the static magnetic field (e.g., the geomagnetism) equals the ratio between the mass and the charge of the respective ion. However, as the authors of the aforementioned review article discuss, it has been hypothesized that EMF in cyclotron resonance frequency alter the physiological activity of the targeted ion. From a physics point of view, however, increasing the kinetic energy of a magnetic field-experiencing ion by its cyclotron frequency can only occur in vacuum or low-pressure gases. In biological microenvironments, by sharp contrast, EMF-induced acceleration of the ion is prevented by damping. Moreover, the restricted dimension of the cellular subcompartment is several thousand orders of magnitude lower than the radius of cyclotron resonance-accelerated ions in vacuum. In addition, the cyclotron frequency refers to dehydrated ion masses (for review see Liboff, 2018). In biological systems, free ions carry a hydration envelope most of the time that may be only shortly shed, e.g., by the selective filter of an ion channel pore during transmembrane transport. Hence, as shown by this example, the molecular basis of the electrobiological energy transfer of weak EMF therapy often remains obscure. In contrast, a more mechanistic understanding has been postulated for the TTFields electrotherapy of cancer as introduced in the second section of this article.

## 2. Tumor treating fields (TTFields), a superweapon against glioblastoma?

TTFields, which have been developed by the company Novocure in Haifa, Israel, are alternating electric fields with intermediate frequency and low intensity. Besides glioblastoma, TTFields therapy is FDA-approved for mesothelioma. In a randomized controlled (but not sham-controlled) clinical trial in newly diagnosed glioblastoma patients (Stupp et al., 2017), patients benefitted from TTFields irrespective of MGMT methylation status of patients. In this trial, TTFields were applied concurrently to temozolomide maintenance therapy (following surgery and adjuvant concurrent temozolomide/fractionated radiation therapy) and compared to temozolomide maintenance therapy alone.

For glioblastoma, alternating electric fields with a frequency of 200 kHz are applied capacitively via ceramic electrode arrays placed onto the shaved scalp of the patient. Modeling the field intensity with due consideration of pre-specified conductivity and relative permittivity of skin, skull and brain tissues has predicted electric field strengths in the range of 1–3 V/cm in the brain tumor (Lok et al., 2019). Table 1 gives an overview of all completed clinical trials of TTFields applications in different cancer entities as of December 12^th^, 2022. Notably, only two trials had active control arms, and among these only NCT00916409 (Stupp et al., 2017) could establish prolonged survival for patients in the TTFields group. Hence, most completed trials so far are not well-versed to inform about the efficacy of TTFields therapy. Further trials should compare TTFields to the current standard of care (SoC) to establish efficacy in other cancer entities as well, which several of the ongoing trials are doing (e.g., NCT03940196 or NCT02973789).

**Table 1:** Overview of clinical efficacy of TTFields in cancer patients.

NCTID	Indication	Intervention	Control group	N	End-point	Intervention	Control	Adverse events related to TTFields	Reference
NCT00749346	NSCLC	NovoTTF-100L + Pemetrexed	-	42	**IFP**	28 weeks	-	mild-moderate dermatitis (14, 33%)	Pless et al. (2013)
					SP	22 weeks	-		
					PR	6 (14.6%)	-		
					SD	20 (48.8%)	-		
					OS	13.8 months	-		
NCT01894061	GBM*	NovoTTF-100A + Bevacizumab	-	25	**PFS**	4.1 months	-	hypertension, cerebral infarct.	[Fallah et al. (2020); abstract^#^]
					**OS**	10.5 months			
NCT02397928	malignant pleural mesothelioma	NovoTTF-100L + Pemetrexed + Cisplatin/Carboplatin	-	80	**OS**	18.2 months	-	skin reactions - grade 1/2: 53 (66%) - grade 3: 4 (5%)	Ceresoli et al. (2019)
					PFS	7.6 months			
					PR	29 (40%)			
					SD	41 (57%)			
NCT02893137	GBM	TTFields + craniectomy	-	15	**toxicity**	-	-	skin reactions grade 1/2 (55%)	Korshoej et al. (2016)
					PFS	4.6 months			
					OS	15.5 months			
NCT00916409	GBM (newly diagnosed)	TTFields + TMZ	TMZ	695	**PFS**	6.7 months	4.0 months	mild-moderate skin toxicity (52%)	Stupp et al. (2017)
					OS	20.9 months	16.0 months		
NCT00379470	GBM (re-current)	NovoTTF-100A	SoC	236	**OS**	6.6 months	6 months	mild-moderate skin toxicity (14% and 2% respectively)	Stupp et al. (2012)
					PFS (at 6 months)	21.4%	15.1%		
					QoL		favored TTFields		

Only completed trials with published results were retrieved from https://clinicaltrials.gov/ and subsequently analyzed. GBM, glioblastoma; IFP, “in field” progression; mo., months; N, patient number; NSCLC, non-small cell lung cancer; OS, overall survival; PFS, progression free survival; PR, partial response; QoL, quality of life; SD, stable disease; SoC, standard of care (chemotherapy); SP, systemic progression; TMZ, temozolomide; *, adult giant cell GBM; adult GBM, adult gliosarcoma; recurrent adult brain tumor; ^#^, only an abstract was available. Endpoints portrayed in bold are primary endpoints of the study.

On the other hand, most studies published so far report few TTFields-specific adverse events, with the exception of mild to moderate skin reactions. Whether this is due to specific electrotherapy effects or due to other effects due to the application itself (frequent scalp shaving, electrode gel, frequent sweating underneath the electrodes) is hard to differentiate without running sham-controlled trials. Last, several trials currently underway analyze TTFields’ efficacy in other tumor entities, such as brain metastases (NCT04967027), gastric- (NCT04281576), pancreatic- (NCT03377491; Rivera et al., 2019), and ovarian cancer (NCT02244502; Vergote et al., 2018).

Preclinical *in vitro* studies have suggested multiple modes of action for the interference of TTFields with tumor biology. TTFields reportedly impair the formation of the spindle apparatus, mitosis and cytokinesis of cancer cells (Kirson et al., 2004; 2009b; Gera et al., 2015; Giladi et al., 2015). Moreover, TTFields have been demonstrated to delay DNA repair (Giladi et al., 2017; Karanam et al., 2017), to inhibit tumor cell motility (Voloshin et al., 2020b) and to permeabilize the plasma membrane of cancer cells (Chang et al., 2018; Aguilar et al., 2021) and the blood brain barrier (Salvador et al., 2023). Furthermore, TTFields reportedly do not impair *ex vivo* viability and function of peripheral blood or glioblastoma-infiltrating T cells and of CAR T-cells (Simchony et al., 2019; Diamant et al., 2021). The tumor preference of these effects has been explained by the proliferation rate that differs between transformed and parental cells. The most effective frequency of the alternating electric fields has been proposed to be determined by the tumor cell geometry (Kirson et al., 2004). TTFields have also been applied to animal tumor models. These preclinical *in vivo* studies confirm TTFields-mediated inhibition of tumor growth (e.g., Grassi et al., 2004), chemotherapy-sensitizing effects (e.g., Giladi et al., 2017), and permeabilization of the blood-brain barrier (Salvador et al., 2022). In addition, TTFields reportedly delay formation of tumor metastases (Giladi et al., 2014a), support the anti-tumor immune response (Voloshin et al., 2020a; Chen D. et al., 2022), and mitigate tumor angiogenesis (Jo et al., 2018).

Mechanistically, TTFields have been proposed to restrict molecular motility of macromolecular dipoles such as tubulin dimers (Kirson et al., 2007; 2009a) and septins (Gera et al., 2015) inhibiting mitotic spindle assembly and correct positioning of the cytokinetic cleavage furrow, respectively. Moreover, condensation of the electric field lines at the cytokinetic furrow and associated dielectrophoresis (i.e., migration of dipoles or charged particles towards higher fields intensities) has been postulated to inhibit symmetric segregation of cellular material to the daughter cells during cytokinesis (Kirson et al., 2004, 2007). As a consequence of these modes of action, TTFields efficacy is dependent on the relative orientation of the cleavage axis of dividing cells to the applied electrical field. Likewise, interference with actin and microtubule dynamics has been suggested to underlie the anti-migratory action of TTFields in tumor cells (Voloshin et al., 2020b) and inhibition of ciliogenesis (Shi et al., 2022). Beyond that, further cell biological effects such as TTFields-triggered replication stress (Karanam et al., 2020) or AMPK-mediated ER stress and autophagy (Shteingauz et al., 2018), have been reported more recently. Combined, these studies point to a highly complex interference of TTFields with several cellular processes.

**Figure 1:**
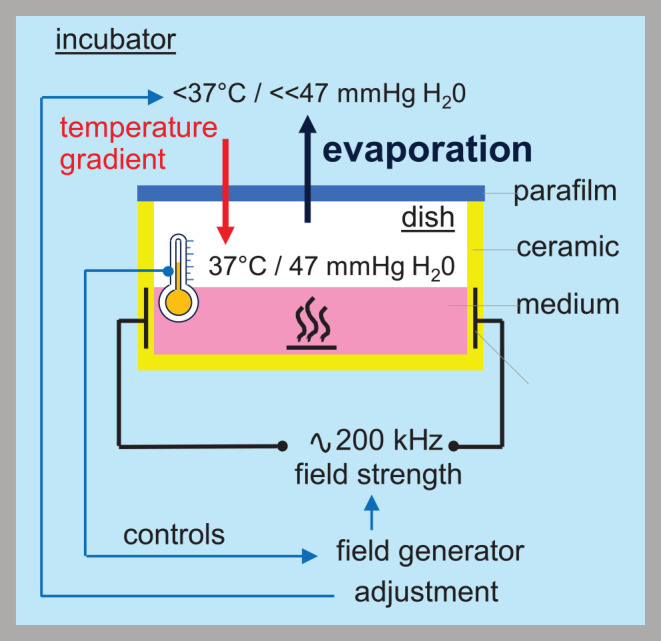
TTFields applied by the Novocure inovitro^TM^ system increase the osmolarity of the medium. Thermal discharge of the inovitro^TM^ system is counter-regulated by decreasing the ambient temperature of the incubator. The field strength is regulated indirectly by adjusting the ambient incubator temperature. Thereby, the temperature of the culture dish is continuously measured and the field strength increased until the dish temperature is equilibrated at 37°C. Different temperatures in incubator and culture dish, however, causes a lower water vapor pressure in the incubator than in the dish, resulting in continuous evaporation from the culture medium.

Notably, the plethora of cellular effects described by *in vitro* experiments might be overestimated due to a misfeature of the inovitro^TM^ system provided by Novocure to study TTFields effects *in vitro*. This device uses ceramic dishes with integrated electrodes for the capacitive injection of the TTFields. The electric fields are delivered parallelly to the cell layer interchangeably from two perpendicular directions in order to increase the number of dividing cells with matching directions of electric field and mitotic cleavage axis. Applying TTFields by the inovitro^TM^ system heats cells and cell culture medium. Field strength, therefore, has to be regulated indirectly: Decreasing the ambient temperature of the incubator below 37°C results by feed-back in an increase in generator power output and TTFields intensity until the cell culture medium and the ceramic dish reach 37°C as controlled by two temperature probes. This indirect control of field intensity, however, harbors the problem that the water vapor pressure in the air atmosphere of the TTFields-heated, 37°C-adjusted ceramic dish (in the better situation sealed with parafilm, in the worst case loosely covered by a plastic lid) exceeds that of the incubator air with lower temperature, resulting in continuous evaporation of water from the culture medium (Figure 1). Drying-out of the cells is prevented by replenishing the lost water volume by adding culture medium. Water evaporation increases and volume replenishment with medium eventually lead to an increase in the osmolarity of the cell culture medium. Since controls are run in 37°C incubators with iso-osmotic cell culture medium, the inovitro^TM^ system in our opinion cannot distinguish between hyperosmotic stress- (Burg et al., 2007; Zhou et al., 2016) and TTFields-mediated cellular effects and hence, the conclusions drawn from the data are limited (discussed also in Slangen et al., 2022). Unfortunately, the majority of the TTFields *in vitro* data were obtained with the inovitro^TM^ system and published without mentioning the system-inherent weakness of the experimental setup (e.g., Voloshin et al., 2016; Chang et al., 2018; Voloshin et al., 2020b; Chen et al., 2022).

Further complicating the picture, a recent theoretical study by Li et al. (2020a) questioned the proposed mechanisms of electrobiological TTFields interactions. The authors concluded that the torsional moment induced by TTFields on dipoles such as tubulin dimers is several orders of magnitude lower than the Brownian energy of the molecules suggesting that TTFields are probably not able to disrupt mitotic spindle assembly. In addition, they estimated for the velocity of particles undergoing dielectrophoresis towards the cytokinetic furrow a value (0.003 μm/s) that is too slow to become biologically relevant during normally ongoing telophase. The same group (Li et al., 2020b) calculated the field distribution for spherical and spindle-shaped cell geometries. These geometries were then used to simulate the TTFields (2 V/cm)-associated temperature distributions for cell models resulting in a negligible TTFields-caused temperature rise. Please note, that this notion is in sharp contrast to the large heat production by the electronically elaborated, high-capacitively field-coupling inovitro^TM^ system. This lack of a significant computed TTFields-mediated temperature rise was directly confirmed experimentally (Li et al., 2020b) suggesting that TTFields effects may not be conferred by hyperthermia.

Consistently, direct temperature measurements of TTFields-treated skin in a melanoma mouse model did not observe TTFields-elicited temperature effects (Li et al., 2016). *Vice versa*, TTFields effect on tumor growth in an ectopic mouse model of pancreatic carcinoma could not be mimicked by 41°C hyperthermia (Castellví et al., 2015). Instead, Li et al. (2020a) proposed a new mechanism for the electrobiological TTFields interaction. Their mathematical modeling predicts a significant TTFields-induced change of membrane potential and the authors hypothesized that this alteration in membrane potential modifies the activity of ion channels. Taken together, the mechanism of electrobiological TTFields transduction still seems to be elusive and modulation of voltage-gated ion channels might be an attractive alternative mechanism. The next paragraph introduces into the biochemistry of voltage sensors, the centerpiece of voltage-gating.

## 3. Molecular bases of voltage-sensing

**Figure 2:**
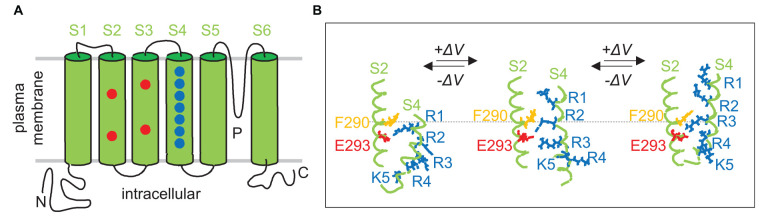
Voltage-sensing at a molecular level. **(A)** Membrane topology of a Shaker voltage-gated (K_v_) K^+^ channel. Drawn is a K_v_ channel alpha subunit with the pore-forming domain (S5, S6, P-loop), the voltage sensor domain with the S4 helix containing basic amino acid residues, and the S2 and S3 helixes with acidic amino acid residues that serve in the voltage sensor as stabilizing counter ions. **(B)** Intramolecular relative movement of voltage sensor during gating. According to the “gating charge transfer center (GCTC) hypothesis”, the aromatic phenylalanine (F290) and the two negative residues E293 and D316 (not shown) of the S2 helix form a GCTC that binds transiently the positive charged residues (blue) of the S4 voltage sensor when consecutively crossing the channel pore area of trans-membrane voltage decline during gating. Scheme shows the deep channel close state at high negative potential (left), and the depolarization of membrane potential-caused successive upward “stepping” of the voltage sensor (middle and right). Transmembrane alpha helixes in **(A,B)** are shown in green, positively charged basic-, negatively charged acidic-, and aromatic (phenylalanine F290) amino acid residues in blue, red, and orange, respectively (redrawn and modified from Catacuzzeno and Franciolini, 2022).

Voltage-gated ion channels such as the shaker K_v_ K^+^- or the L type Ca_v_ Ca^2+^ channel activate upon depolarization of the membrane potential. The general construction principle of these channels is composed of four times six lipophilic transmembrane alpha helices (S1–S6) either realized by tetramers of four alpha subunits (K_v_; Jiang et al., 2003) or a single alpha subunit with 24 transmembrane domains (Ca_v_; Catterall et al., 2005). The four S5 and S6 helices together with the inter-connecting P loops form the ion-conducting pore and determine the ion specificity while the four S1-S4 helices around the S5–S6 helix bundle create the voltage sensor. The S4 helix contains several (typically four) positively charged amino acid residues (mostly arginine) separated from each other by two uncharged residues. These positively charged residues that are referred to as gating charges, are located within the membrane and become electrostatically stabilized, e.g., by negatively charged amino acids (i.e., countercharges) in the other (S1–S3) helixes of the voltage sensor (for review see Catacuzzeno and Franciolini, 2022; Figure 2A).

The voltage sensor (S1–S4) can be envisaged as a gating channel with the S4 helix inside this channel. According to the sliding helix model of the voltage sensor action (Yarov-Yarovoy et al., 2012; Catterall et al., 2017), the S4 helix is pulled in towards the cytoplasmic membrane face at negative resting membrane potential. Membrane depolarization decreases this electrostatic force and the S4 helix is dragged out by a few Å towards the external membrane face thereby initiating structural changes in the S5–S6 helix bundle eventually leading to ion channel activation. Structurally, the gating channel has been proposed to be shaped as an hourglass-like pore with two crevices reaching from in- and outside, respectively, deeply in the membrane. A hydrophobic, by a phenylalanine residue-built, central plug (hydrophobic constriction site) isolates the two crevices electrically from each other. As a result, the whole membrane voltage drops across the central plug along a few Å membrane distance (for review see Catacuzzeno and Franciolini, 2022).

Multiexponential fitting and noise analysis of gating currents, structural information obtained by, e.g., X-ray crystallography or cryo-electron microscopy, simulation of molecular dynamics and other approaches suggest that the voltage-dependent sliding of the S4 helix sequentially proceeds by intermediate steps “between free energy basins separated by rate-limiting free energy barriers” (Delemotte et al., 2017). The former result from ion pair or hydrogen-bridge bonds of the gating charges with their respective countercharges or polar phospholipid head groups and other ionic or lipophilic interactions within the three-dimensional molecular environment of the voltage sensor. By consecutively passing the central plug, the gating charges stride through the voltage gradient thereby generating a gating current (12–14 *q*_e_ elementary charges) that can be directly analyzed electrophysiologically (when the subsequently gated ion currents through the four S5-S6 helix bundle-built ion channel pore are prevented experimentally; for review see Catacuzzeno and Franciolini, 2022). A slightly modified model of voltage gating, the gating charge transfer center (GCTC) hypothesis, is described in Figure 2B in more detail.

Notably, cryo-electron microscopy and molecular dynamic simulation have revealed structural-functional differences between the four voltage sensors of the rabbit L-type Ca_v_ 1.1 Ca^2+^ channel. While, for instance, the S4 helix of the voltage sensor domain-I sequentially proceeds through three macrostates (resting 1–2 and activated state) with long transition times in the high μs to low ms timescale, that of the voltage sensor domain-IV steps through four macrostates (resting 1–3 and activated state) with transition times in the sub-μs to low μs timescale (Fernández-Quintero et al., 2021). Combined, these data suggest complex transitions between conformational macrostates of the Ca_v_1.1 voltage sensor domains that exhibit differing kinetics and energy barriers. As a consequence thereof, one might assume that: (i) channel activation can not only be triggered by intrinsic membrane depolarization (due to, e.g., inactivation of K^+^-permeable- or activation of Na^+^-permeable ion channels) but also by external low intensity EMFs, and (ii) because of the multi-kinetics process of gating, transfer of activation energy from external EMFs may occur at several EMF frequencies (see also section 6 “Are 200 kHz TTFields too high-frequency for ion channel modulation in glioblastoma cells?” for a more detailed discussion on this issue). As a matter of fact, simulation of molecular dynamics unraveled that voltage sensors of voltage gated ion channels are prone to be directly affected by external electromagnetic fields. In particular, very short pulsed electric fields have been forecasted to activate voltage-gated ion channels while longer pulses, as used for electroporation of the plasma membrane, are predicted to convert the gating channels of the voltage sensor domain into a transient or a more complex phospholipid headgroup-stabilized long-living pore (Rems et al., 2020). Strong evidence for such a high susceptibility of voltage-gated ion channels to low intensity EMFs can be found in several fish species, which are capable of sensing very low electric fields with voltage-gated ion channels as signal transducers, as illustrated in the next paragraphs.

## 4. Biological voltmeters

**Figure 3:**
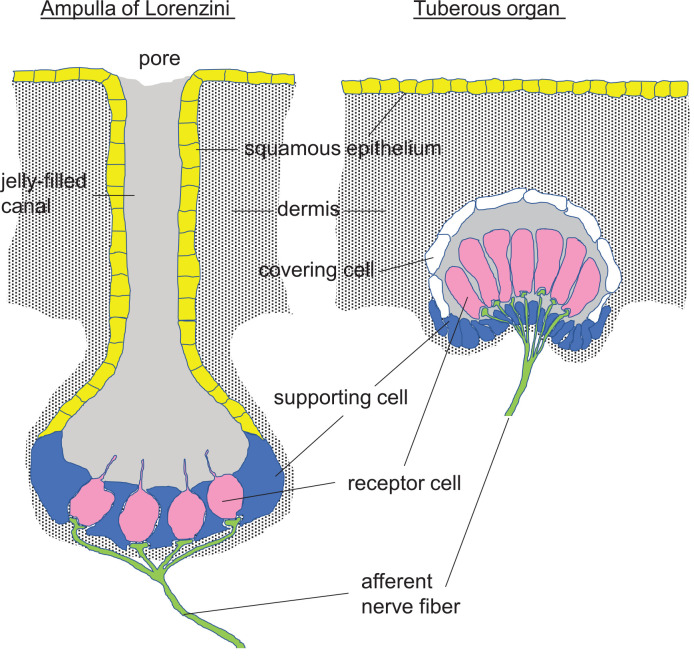
Perception of electric fields by fishes conductively and capacitively coupled to the electrosensory receptor cells (pink) in an Ampulla of Lorenzini and a Tuberous organ, respectively [redrawn and modified from Helfman et al. (2009) and Baker (2019)].

**Figure 4:**
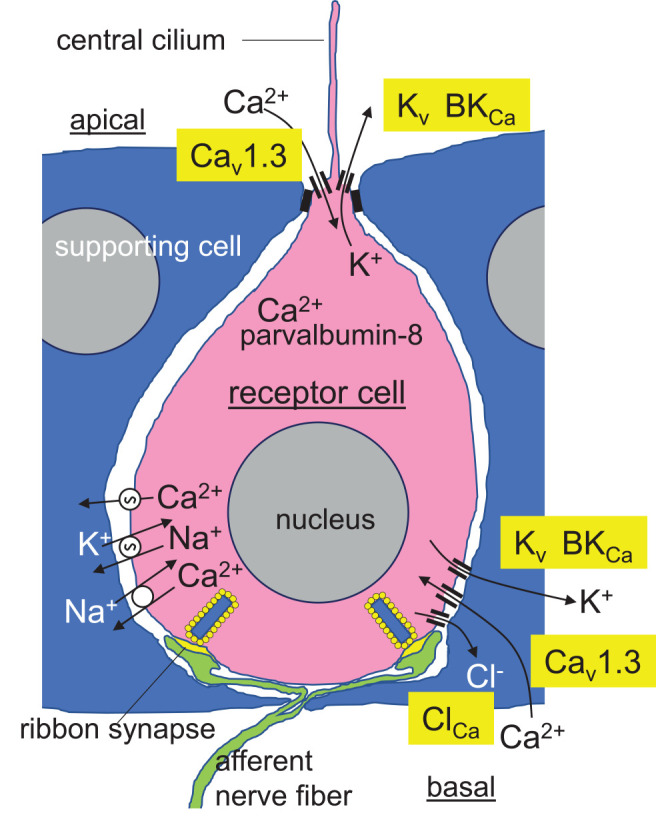
Hypothetical model of a receptor cell (pink) in the epithelium of the skate ampullae of Lorenzini. Tight junctions of the encompassing supporting cells (blue) with the receptor cells electrically isolate the high-resistance apical membrane of the receptor cells from the basolateral interstitial space. Low intensity electric fields modify the activity of Ca_v_1.3 L-type voltage-gated Ca^2+^ channel in the apical membrane and central cilium resulting in membrane depolarization of the apical membrane and apical Ca^2+^ entry. Depolarization is conducted to the basal membrane followed by activation of probably basal Ca_v_1.3 Ca^2+^ channels, basal Ca^2+^ entry, amplification of the membrane depolarization and Ca_v_1.3 activity by Ca^2+^-activated Cl^−^ channels, and transmitter release from the ribbon synapses. Apically entered Ca^2+^ is buffered by the Ca^2+^-binding protein parvalbumin-8, limiting the activity of apical Ca^2+^-activated BK K^+^ channels, which repolarize the basal membrane and maintain it close to the activation voltage threshold of the Ca_v_1.3 channels. Alternating activity of Ca_v_1.3 and basolateral BK- or/and voltage-activated K_v_ K^+^ channels programs oscillation of basal membrane potential and free cytosolic Ca^2+^ concentration with synchronized transmitter release. Ca^2+^ is extruded across the basal membrane by Ca-ATPase and Na/Ca-antiporter (from Clusin and Bennett, 1977a, b; Clusin and Bennett, 1979a, b; Lu and Fishman, 1995; Graydon et al., 2011; Bellono et al., 2017; Baker, 2019; Clusin et al., 2019; modified).

Passive or active electrolocation is realized in species of cartilaginous and bony fishes. In passive electrolocation, fishes perceive weak electric fields that are generated by the neuronal, muscle, or gills ion pump activity of other animals and that are conductively coupled to specialized organs such as the ampullae of Lorenzini in elasmobranch fishes (Figure 3). The elephantfish (*Mormyridae*), an example of a species that is capable of active electrolocation, generates weak alternating electric fields with its electroorgan in the tail and analyzes distortions of the field that is coupled capacitively to tuberous organs (knollenorgans) in the skin (Figure 3). The ampullae of Lorenzini consist of electrosensory receptor- and auxiliary supporting cells lining a cavity beneath the skin that is conductively connected to the skin surface by canals. These canals are filled with seawater-containing jelly, have electrically resistant luminal cell surfaces and cell-cell connections, and thus, act as electrical wires that conduct the electric fields to the apical membrane of the electrosensory receptor cells. The epithelium of the cavity may contain thousands of these receptor cells that are surrounded by supporting cells. Epithelial conductivity of supporting cells, as well as paracellular shunts between supporting cells or supporting and receptor cells, seems to be lower than that of the receptor cells. The latter are cone-shaped with a large basal cell pole and a very small apical membrane area that projects a central kinocilium into the cavity lumen. In receptor cells, the apical membrane exhibits a severalfold higher electrical resistance than the basal membrane leading to a steeper potential drop of transepithelial voltage across the apical than basal membrane (Clusin and Bennett, 1977a, b). Electric fields have been shown to activate voltage-gated Ca^2+^ channels in the apical membrane which lead to Ca^2+^-influx-mediated membrane depolarization and subsequent activation of adjacent Ca^2+^- (skate) or voltage-activated (catshark) K^+^ channels in the apical membrane (Leitch and Julius, 2019; Figure 4).

Electrophysiological and receptor cell transcriptome analyses in skate receptor cells have demonstrated high expression of orthologs of the mammalian L-type Ca_v_1.3 (*KACNA1D*) voltage-gated Ca^2+^ channel and the BK_Ca_ Ca^2+^-activated K^+^ channel in the apical membrane and kinocilium of the electrosensory receptor cells [Bellono et al., 2017; and in preprint (Chen A. L. et al., 2022)]. Ca_v_1.3 of skate receptor cells reportedly exhibits a lower threshold (i.e., the activation occurs at more negative voltages), a steeper activity/voltage relationship (i.e., during membrane depolarization maximal activity is immediately reached after passing the threshold of activation), and a slower inactivation kinetic as compared to its mammalian counterpart. Moreover, Ca_v_1.3-mediated Ca^2+^ entry and subsequent activation of adjacent Ca^2+^-activated BK_Ca_ K^+^ channels do not result in strong membrane hyperpolarization (and complete deactivation of Ca_v_1.3) of receptor cells since receptor cells express high amounts of Ca^2+^ buffering parvalbumin-8 protein and Ca^2+^-ATPase that, both, instantaneously decrease cytosolic free Ca^2+^ concentration, and thus, dampen BK_Ca_ channel activity (Figure 4). As a consequence, apical membrane potential of receptor cells is maintained around the activation threshold of Ca_v_1.3 generating an extremely sensitive system that transduces external electric fields in Ca^2+^ action potentials (Bellono et al., 2017). Apical membrane depolarizations are conducted to the basal membranes of these secondary sensory receptor cells that form ribbon synapses with afferent fibers. Ca^2+^-activated Cl^−^ channels and consecutive Cl^−^ efflux probably amplify the depolarizing signals at the basal membrane (Lu and Fishman, 1995) triggering activation of basal L-type voltage-gated Ca^2+^ channels such as basally located Ca_v_1.3 channels (Chen A. L. et al., 2022) followed by activation of basal K^+^ channels including BK_Ca_ channels (Clusin et al., 2019; Chen A. L. et al., 2022). Ca^2+^ is extruded across the basal membrane by Ca-ATPase and Na/Ca-antiporter. The latter is powered by the inwardly directed electrochemical gradient of Na^+^ as maintained by the Na/K-ATPase (Figure 4). The interplay of these channels/transporters/pumps results in oscillation of the basal membrane voltage and accompanying oscillation of the cytosolic free Ca^2+^ concentration (Clusin et al., 2019). Under resting conditions, these oscillations are thought to trigger basal transmitter release and afferent neural activity in a synchronized manner (Bellono et al., 2017). Convergent connectivity of the afferent neurons further adds sensitivity to the system (Peters et al., 1997).

Reportedly, electric field strengths as low as 1 nV/cm can be perceived. The ampullae of Lorenzini have been proposed to detect slow changes (frequencies between 0.1 and 50 Hz) in electric field strength (for review see Crampton, 2019) with probably highest sensitivity at the resonator frequency of the receptor cell (see also section 6 “Are 200 kHz TTFields too high-frequency for ion channel modulation in glioblastoma cells?”). In addition to Ca_v_1.3 and BK_Ca_, high expression of the orthologs of the mammalian K_v_1.1 and K_v_1.5 voltage-gated K^+^ channels in the receptor cells has been deduced from skate transcriptome data (Clusin et al., 2019), suggesting a more complex interplay of voltage-dependent ion channels in the voltage/Ca^2+^ transduction process (Figure 4).

Likewise, the orthologs of mammalian K_v_1.x-like voltage-gated K^+^ channels are reportedly expressed by tuberous organs that are used for active electrolocation of, e.g., elephantfish (Smith et al., 2006). These tuberous electroreceptors are electrically isolated from the environmental fresh water. External electric fields, therefore, are capacitively coupled to the receptor cells that respond to AC electric organ discharges in the stimulus range of 20 Hz-18 kHz (25 kHz; for review see Kramer, 2009; Crampton, 2019; Figure 3). To the best of our knowledge, the molecular mechanism of electroreception in tuberous organ cells has not been defined, yet. Although tuberous organ and ampullae of Lorenzini evolved in a non-homologous manner in phylogenesis, it is tempting to speculate that tuberous organs also employ voltage-gated ion channels for electro/biochemical transduction.

In summary, specialized organs are capable of detecting electric field strengths that are several orders of magnitude lower than the fields applied in, e.g., anti-cancer electrotherapy. Moreover, voltage-gated ion channels have been identified at least in the ampulla of Lorenzini to act as signal transducers. Given the tight voltage reliance of voltage gated-ion channels, which has been phylogenetically tuned up to an ultra-high voltage sensitivity in electroreceptors of, e.g., fishes as compared to the very high field strengths applied in electrotherapy, it is intriguing to speculate that externally applied electromagnetic fields, such as in electrotherapies, indeed directly modify the conformational transitions of the voltage sensor domains in voltage-gated ion channels resulting in modified ion channel activity. Actually, several studies reported ion channel modulation by external electromagnetic fields as summarized in the next paragraphs.

## 5. Ion channels as targets of low frequency (<1 kHz) electrotherapy

Many carcinoma entities (Brackenbury, 2012; Djamgoz et al., 2019), as well as gliomas (Xing et al., 2014), upregulate voltage-gated Na^+^ (Na_V_) channels that are required for the function of excitable cells in normal tissue, such as neurons or muscle cells. In carcinoma resection specimens, Na_v_ channel abundance reportedly is associated with formation of distant metastases and poorer patient survival. Proposed cellular mechanisms comprise Na_v_ activity-regulated H^+^ extrusion *via* allosterically coupling to Na/H exchangers (NHE) at the lamellipodium of tissue-invading cancer cells. Local extracellular acidification, in turn, increases activity of cathepsins and metalloproteinases and promotes tissue invasion (Luo et al., 2020). Static (direct current, 0 Hz) electric fields (DC-EF, 0.1–4 V/cm) have been shown *in vitro* to direct migration of highly migratory (but not of weakly migratory) prostate cancer cells towards the cathode. This “galvanotaxis” was blocked by the Na_v_ blocker tetrodotoxin suggesting a function of Na_v_ channels in the electrobiological transduction. The epithelia of the prostate gland generate a transepithelial voltage of about -10 mV measured at the luminal as compared to the earthed basolateral side. The Na_v_-dependent “galvanotaxis” alongside this physiological transepithelial voltage gradient that corresponds to an electric field intensity of 5 V/cm has been hypothesized to promote invasion of prostate cancer cells into the gland ducts and metastasis (Djamgoz et al., 2001). Moreover, DC-EF (1–3 V/cm for 15 min) reportedly stimulates Ca^2+^ entry, firing rate, and insulin secretion in mouse insulinoma (bTC-6) β cells (Liebman et al., 2021). This DC-EF effect was sensitive to verapamil, a blocker of L- and T-type voltage-gated Ca^2+^ Ca_v_ channels (Bergson et al., 2011) suggesting an involvement of Ca_v_ channels in the transduction of the static electric field into Ca^2+^ signaling.

Pulsed EMF (PEMF) have been demonstrated *in vitro* to stimulate osteogenesis (Petecchia et al., 2015; Benya et al., 2021; Kar et al., 2021; He et al., 2022). Polycystin-2 (TRPP2, *PKD-2*), a voltage-dependent Ca^2+^-permeable cation channel of the polycystic subfamily of the transient receptor proteins (TRP), harbors a voltage sensor domain similar to other voltage-gated ion channels (Shen et al., 2016) and is expressed in rat osteoblasts. In these cells, TRPP2 is located on the primary cilium where it reportedly transduces PEMFs (0.6 mT, 50 Hz) into biological signaling that promotes osteogenic differentiation as evident from TRPP2 knock-down and pharmacological targeting (He et al., 2022). Consistent with a pivotal role of Ca^2+^ signaling in PEMF-stimulated osteogenesis, early osteogenic differentiation of human bone marrow stroma-derived mesenchymal stem cells by PEMF (2 mT, 1.3 ms pulses at 75 Hz for 10 min/day and several weeks) was paralleled by a continuously increasing steady state free cytosolic Ca^2+^ concentration and upregulation of L-type Ca_v_ channel expression (Petecchia et al., 2015). Similarly, PEMF (1 mT, 50 Hz)-induced neuronal differentiation of cultured dorsal root ganglion neurons has been shown to depend on L-type Ca_v_ channel-triggered Ca^2+^ signaling (Li et al., 2014). Along those lines, ELF-EMF (100 Hz for 120 h) co-administered with temozolomide has been demonstrated, beyond increasing Ca^2+^, to stimulate downregulation of stem cell and upregulation of differentiation markers in human glioblastoma U87 cells (Ahmadi-Zeidabadi et al., 2019). An increase in steady state free cytosolic Ca^2+^ concentration following PEMF (3 mT, 50 Hz) treatment has also been demonstrated in a human astrocyte cell line (Aldinucci et al., 2000). Finally, nano- to picoseconds-pulsed PEMF using electric fields of high (kV/cm) voltage have been demonstrated *in vitro* to activate Ca_v_ and other channels in various cell types (Craviso et al., 2010; Semenov et al., 2015; Burke et al., 2017; Azarov et al., 2019). In two of these studies, Ca_v_ channel activation secondarily to electroporation-mediated membrane depolarization was the most likely mechanism (Azarov et al., 2019) and could not be excluded (Craviso et al., 2010), respectively.

Extremely low-frequency electromagnetic fields (ELF-EMF, 8 Hz for 48 h) reportedly stimulate Ca^2+^ influx in B16-F10 melanoma cells that is blunted by L- and T-type Ca_v_ channel blockers (Wang et al., 2021). Functionally, ELF-EMF (1 mT, 100 Hz 1.3 ms, 2 h/day for 5 days) have been demonstrated to induce cell death in MC4-L2 breast cancer cells in a verapamil-sensitive manner (Barati et al., 2021). Likewise, a growth-impairing action of ELF-EMF (5–10 μT, frequency-modulated in a 25–6 Hz and 6–25 Hz frequency pattern, 1 h/day for 3–5 days) has been demonstrated in HeLa (cervix carcinoma), MDA-MB-231, and MCF-7 (both breast carcinoma), as well as B16-BL6 (mouse melanoma) cells but not in three non-malignant cell lines (Buckner et al., 2015). In this study, ELF-EMF (>15 min) induced in HeLa, MDA-MB-231, MCF-7, and B16-BL6 an increase in cytosolic free Ca^2+^ that was prevented by preincubating the cells with the Ca_v_ blockers bepridil or mibefradil. In the only cell line tested (B16-BL6), T-type Ca_v_ channel inhibition abolished the ELF-EMF-induced impairment of proliferation (Buckner et al., 2015). In sharp contrast, pro-proliferative and anti-apoptotic actions of ELF-EMF (50 Hz, 1 mT) have been shown in human neuroblastoma IMR32 and rat pituitary GH3 cells, which were sensitive to the L-type Ca_v_ blocker nifedipine. Here, ELF-EMF induced Ca_v_ expression but not activity (Grassi et al., 2004) suggesting that Ca_v_ channels are ineligible for electrobiological signal transduction. Besides growth modulation, ELF-EMF (0.7 mT, 60 Hz, 2 × 2 h/day for 7 days) reportedly promote neurite outgrowth of cultured rat chromaffin cells that is blocked by nifedipine (Verdugo-Díiaz et al., 2002).

Beyond Ca^2+^ channels, ELF-EMF (268 μT and 902 μT, 20 Hz) have been demonstrated to stimulate voltage-gated K_v_1.3 (*KCNA3*) K^+^ currents of K_v_1.3-expressing Chinese hamster ovary (CHO) cells in patch-clamp-whole-cell recording. Here, K_v_1.3 activation occurs few seconds after switching-on ELF-EMF exposure and lasted several minutes after field removal (Cecchetto et al., 2020). Combined, these data strongly suggest that external low intensity, low frequency EMFs exert at least part of their biological *in vitro* effects by modulation of voltage-gated ion channels. That similar mechanisms are also applying for external low intensity EMF therapies in the intermediate and high frequency range (such as TTFields application in glioblastoma), in contrast, is much harder to imagine. The obvious reason for that is the kinetics of biological excitability which seems to be not fast enough to respond to intermediate/high EMF frequencies. As introduced in the next paragraphs, auditory hair cells are well suited to estimate the kinetics and frequency limitations of biological sensory responses and to get some insights into possible mechanisms of TTFields effects in glioblastoma.

## 6. Are 200 kHz TTFields too high-frequency for ion channel modulation in glioblastoma cells?

In frogs, turtles, lizards or birds, hair cells may exhibit electrical auditory tuning that contributes to the spectrum analysis of the acoustic frequency band. Thereby, individual hair cells differ in their electrical resonance frequency, i.e., in the acoustic (or experimentally applied electrical stimulus) frequency where they are responding best with synchronous oscillation of the membrane potential and transmitter release. Mechanistically, the acoustic activation of transducer channels in the apical membrane of the hair cell induces a membrane depolarization that stimulates in the basolateral membrane voltage-gated Ca_v_ Ca^2+^ channels. This results in further membrane depolarization, Ca^2+^ entry, and subsequent activation of adjacent BK_Ca_ Ca^2+^-activated and/or voltage-gated K_v_ channels very similar to the situation in the receptor cells of the ampullae of Lorenzini (see section 4 “Biological voltmeters”). Activation of K^+^ channels re(hyper)polarizes the membrane potential leading to deactivation of the Ca_v_ Ca^2+^ and K_v_ K^+^ channels, to a decrease of the cytosolic free Ca^2+^ concentration, and consequently to deactivation of the BK_Ca_ K^+^ channels. The transducer channel-triggered consecutive activation and deactivation of Ca^2+^ and K^+^ channels oscillate the membrane potential in an acoustic frequency-synchronous manner (for review see Fettiplace and Fuchs, 1999).

In electrically tuned hair cells, this frequency is constrained to a narrow resonance frequency band which is determined by the size and interconnected membrane capacitance of the hair cell and the number and kinetics of basolateral Ca^2+^ and K^+^ channels. Especially the kinetics of the expressed BK_Ca_ splice variants and auxiliary beta sub-units limit the maximal frequency a hair cell is electrically tuned at. In poikilothermic animals, this frequency range, as measured at room temperature or below, is reportedly limited to 1 kHz [with a minimal documented value of 250 Hz for the bull-frog *Rana catesbeiana* (Hudspeth and Lewis, 1988)]. In birds, the maximal resonance frequency increases with a Q_10_ = 2 at higher temperatures suggesting a maximal frequency of membrane potential oscillation up to 4 kHz in electrically tuned avian hair cells (for review see Fettiplace and Fuchs, 1999). Likewise, mammalian auditory inner hair cells, which do not show electrical tuning, may generate membrane oscillations synchronous to the acoustic stimulus with frequencies up to 2–3 kHz as demonstrated among others in guinea pig (Palmer and Russell, 1986).

As already mentioned, acoustically elicited oscillation of membrane potential encompasses several serially occurring time-dependent processes. These comprise beyond serial activation/deactivation of the involved channel variants, cyclic re-charging of the apical and basolateral membrane according to intrinsic capacitance-determined time- and resistance-dependent length constants. As a consequence thereof, the actual time constants of the subsequently occurring sub-processes, such as activation of the Ca_v_ Ca^2+^ channels in the basolateral membrane, must be profoundly shorter than an entire oscillation period of the membrane potential. As a matter of fact, in a patch-clamp study on auditory hair cells of chicken, activation of L-type voltage-gated Ca_v_ Ca^2+^ channel-generated Ba^2+^ currents occurred with a (voltage step-dependent) time constant down to *τ* = 100 μs (Zidanic and Fuchs, 1995). One has to bear in mind that this experimentally deduced time constant reflects also “non-biological” time-consuming processes. The latter includes patch-clamp amplifier-intrinsic, patch-pipette resistance-, as well as pipette and membrane capacitance-caused time delays of voltage clamping (Zidanic and Fuchs, 1995). Thus, the “real” activation kinetics of the studied Ca_v_ Ca^2+^ channel might be even faster. Along those lines, ion channels involved in fish electric organ discharge frequencies in the 20 kHz range (Kramer, 2009, Crampton, 2019; see section 4 “Biological voltmeters”) must operate with faster activation time constants than 100 μs. Notwithstanding, the presumed kinetics of voltage-gated ion channels in those very fast biological systems (auditory hair cells, electric organs, etc.) still seems to be too slow to be “in resonance” with the 200 kHz EMF of TTFields applied in glioblastoma therapy.

RT-PCR data of two human glioblastoma cell lines (T98G and U251; Neuhaus et al., 2019) as well as transcriptome data of glioblastoma resection specimens deposited in the TCGA database (Vatter et al., 2020) suggest high expression of Ca_v_1.2 (*CACNA1C*) and Ca_v_1.3 (*CACNA1D*) L-type voltage-gated Ca^2+^ channels. Moreover, patch-clamp recordings in human glioblastoma cell lines T98G (Steinle et al., 2011; Stegen et al., 2015), U-87MG (Edalat et al., 2016), U251 (Stegen et al., 2016) and RT-PCR data from primary cultures of mesenchymal glioblastoma stem cells suggest high (functional surface) expression of BK_Ca_ K^+^ channels (Ganser et al., 2022). In this way, glioblastoma cells resemble strikingly auditory hair cells or receptor cells in the Ampullae of Lorenzini. It is, therefore, intriguing to speculate that glioblastoma cells might show a certain degree of electrical “excitability” and resultant vulnerability to external EMFs. Along those lines, glioblastoma cells have been demonstrated to neuro-mimic neurogenesis programs (Venkataramani et al., 2022) and to integrate into neuro-glial networks by synaptogenesis (Venkatesh et al., 2019). In addition, they have been proposed to pursue oscillation of cytosolic Ca^2+^ and membrane potential to execute programs of brain invasion (Catacuzzeno and Franciolini, 2018).

Our previous study on single glioblastoma cells (human T98G and U251 cell lines, Neuhaus et al., 2019) demonstrated that TTFields (200 kHz, 0.25–2.5 V_pp_/cm) induce a field strength-dependent increase in cytosolic free Ca^2+^ concentration in Fura-2 Ca^2+^ imaging experiments. Notably, this increase was not due to simple membrane electroporation since Ca_v_ Ca^2+^ channel blockers abolished TTFields-stimulated increase in cytosolic free Ca^2+^ reversibly. Moreover, the knock-down of Ca_v_1.2 blunted this effect (Neuhaus et al., 2019). Furthermore, in cell-attached patch-clamp experiments with 2.5 V_pp_/cm TTFields, this Ca^2+^ increase was paralleled by activation of BK_Ca_ K^+^ channels. Both, TTFields-stimulated BK_Ca_ and Ca_v_ channel activities were long-lasting and outlasted withdrawal of TTFields application by several minutes (Neuhaus et al., 2019). Notably, an identical time course has been observed for ELF-EMF-stimulated activation of voltage-gated K_v_1.3 K^+^ currents in CHO cells (Cecchetto et al., 2020), as already mentioned above in Section 5 “Ion channels as targets of low frequency (<1 kHz) electrotherapy.”

Furthermore, we found that TTFields (200 kHz, 1 V_pp_/cm) induced slight perturbations of cell cycle progression and dissipation of inner mitochondrial membrane potential. This resulted in small but significant cell death and a decrease in clonogenic survival in one (T98G) of the two glioblastoma cell lines tested (Neuhaus et al., 2019). Unexpectedly, inhibiting Ca_v_ channels by benidipine was unable to revert these TTFields effects suggesting that Ca_v_ channel activity did not contribute to the apparent tumoricidal action of the EMF in T98G cells. Rather, benidipine exerted effects slightly additive to those of the TTFields (Neuhaus et al., 2019). This might suggest that the TTFields-stimulated Ca_v_ channel activity and subsequent rise in cytosolic free Ca^2+^ concentration not necessarily must have detrimental consequences for the affected tumor cell.

**Figure 5:**
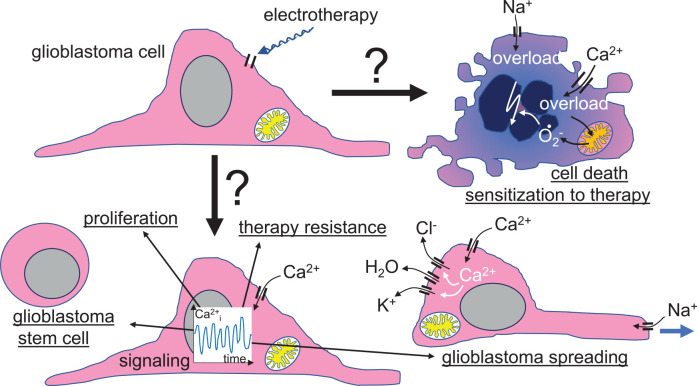
Speculative scenario of a primary voltage-gated ion channel targeting by glioblastoma electrotherapy. Beyond tumoricidal or therapy-sensitizing effects, electrotherapy-elicited modulation of ion channel activities might also boost malignant progression of glioblastoma cells by inducing transition in cancer stem cell phenotypes, cell proliferation, therapy resistance, and/or cell migration, glioblastoma spreading, and brain invasion.

Combined, the data described in sections 5 “Ion channels as targets of low frequency (<1 kHz) electrotherapy” and 6 “Are 200 kHz TTFields too high-frequency for ion channel modulation in glioblastoma cells?” demonstrate that low intensity EMF may interfere with cellular Ca^2+^ signaling in a wide range of frequencies including the 200 kHz frequency of the TTFields therapy. In the case of TTFields, one might speculate that the putative discrepancy between the reported kinetics of voltage-gated channels and the 200 kHz of TTFields might be reconciled by considering the multistep nature of voltage gating as described in section 3 “Molecular bases of voltage-sensing”: TTFields might interfere with sub-stepping between conformational macro states of the activating/deactivating channels that occur with much faster kinetics than the complete activation/deactivation process. Following the “ion channel hypothesis”, the reported tumor specificity of TTFields therapy (Kirson et al., 2007; Chang et al., 2018) must then arise from a tumor-specific expression/upregulation of TTFields-vulnerable types or variants of voltage-gated channels. As an example, glioblastoma cells have been demonstrated to express a specific splice variant (_glioma_BK_Ca_) of BK_Ca_ channel that exhibited highest Ca^2+^ sensitivity among the (at that time) known BK_Ca_ variants (Liu et al., 2002).

Another conclusion of the above-described studies is that the reported cellular EMF responses are cell type-specific and may span anti-proliferative and pro-apoptotic, pro-proliferative and anti-apoptotic, or phenotype-(de)differentiating effects. Moreover, the majority of the above-mentioned studies have identified voltage-gated ion channels and, in particular, Ca_v_ channels as primary EMF targets, strongly suggesting these channels as biological antennas of weak external electromagnetic fields.

## Concluding remarks

In accordance with the tenor of the present review article, several authors have proposed voltage-gated (dependent) ion channels as molecular transducers of weak external EMF fields into biological signals (e.g., Pall, 2013, 2015, Pall, 2016; Li et al., 2020a; Georgiou and Margaritis, 2021; Panagopoulos et al., 2021; Wust et al., 2021). Identification of voltage-gated ion channels as central functional units in electrolocation of fishes (see section 4 “Biological voltmeters” of this article) may further support this “ion channel hypothesis” of external EMF perception. The wide range of EMF frequencies that comprise static fields (0 Hz) as well as radio frequency (MHz) and that reportedly all evoke activity changes of ion channels might be explained by the multi-step kinetics of ion channel gating with several “resonance” frequencies in this process (see section 3 “Molecular bases of voltage-sensing” of this article).

Although biological effects of weak external EMF have been demonstrated under controllable *in vitro* and preclinical *in vivo* conditions, their clinical relevance remains still highly debatable. Biological organisms are electrically active and life evolved in an ever-changing electromagnetic microenvironment due to the switch-on/switch-off activity of muscles or neurons in higher animals or locomotion-caused re-orientation within the terrestrial magnetic field. Bearing that in mind, one can assume that cells are well adapted to weak external EMF. This point is underlined since artificial EMF emitted by our electric devices of daily use (e.g., 50–60 Hz power supply, WiFi, cell phone, radio, TV, microwave, etc.), when used according to the guidelines and within the administrative limits of field strengths, seem not to elicit gross biological responses (Vijayalaxmi and Scarfi, 2014). Hence, strong effects of EMF in electrotherapies should not be expected. Nevertheless, electrotherapies have raised large hopes, especially in cancer entities with bad prognoses, such as glioblastoma. In this case, the hope is fueled by case reports on TTFields-associated complete tumor remission (Kessler et al., 2020; Stein et al., 2020) and one randomized controlled clinical trial (Stupp et al., 2017). As described above, more randomized controlled trials with proper control arms are underway to prove the replicability and generalizability of TTField’s effects against cancer.

Neoplastic transformation and malignant progression have been shown to rely on a remodeling of the ion channel toolkit of the tumor cell (Huber, 2013). In particular, many tumor entities upregulate voltage-gated channels like Na_v_ (Martin et al., 2015), K_v_ (Pardo and Sühmer, 2008; Lastraioli et al., 2015), or Ca_v_ (Morrone et al., 2016) channels. Since these “oncochannels” reportedly play pivotal roles in tumor biology, electrotherapy may also entail risks when altering activities of these channels. Human glioblastoma cell lines, for example, have been shown to respond to weak DC-EFs with voltage-gated channel-dependent electrotaxis (Tsai et al., 2020) which might hint at the possibility that EF stimulates glioblastoma brain spreading. Moreover, EF reportedly induce a Ca_v_-dependent proliferation and neuronal differentiation of neural stem cells (Wang et al., 2023) indicative of potential beneficial effects of EF therapies on neural tissue. EMF may also induce resistance against apoptosis in brain tumor cells (Grassi et al., 2004). Along those lines, in our previous work, TTFields-induced activation of L-type voltage-gated Ca_v_1.2 Ca^2+^ channels in one human glioblastoma cell line exerted rather pro-survival (Neuhaus et al., 2019) than tumoricidal effects (Figure 5).

Therefore, a better understanding of the functional significance of potential electrobiological transducer channels in tumor biology and therapy resistance (for reviews see Huber, 2013; Klumpp et al., 2016; Roth and Huber, 2022) is inevitable for the development of further strategies in cancer electrotherapy. Such future strategies might aim to augment electrotherapy-induced cellular stress or to suppress cellular resistance mechanisms by concomitant targeting of potential resistance-mediating transducer channels. Moreover, once the electrobiological transduction process and the involved channel subtypes/splice variants are disclosed, electrotherapy protocols (waveform/frequency/intensity/duration/pulsed vs. continuous application) might become easily optimized in e.g., heterologous expression systems. Finally, the identification of potential electrotherapy transducer channels in a certain tumor entity may be utilized for personalization of electrotherapy, e.g., by stratification of patients according to the abundance of the transducer channels in the tumor resection specimens. However, to ultimately achieve this, much more joint efforts of electrotherapy developers, oncologists, physiologists and physicists are required.

## Author contributions

TA: conception and design. All authors: literature search, writing, and review of the manuscript. All authors contributed to the article and approved the submitted version.

## Funding

FE and SH received funding from the German Research Foundation (DFG EC 575/2-1 and HU 781/7-1).

## Conflict of Interest

The remaining authors declare that the research was conducted in the absence of any commercial or financial relationships that could be construed as a potential conflict of interest.

## Publisher’s note

All claims expressed in this article are solely those of the authors and do not necessarily represent those of their affiliated organizations, or those of the publisher, the editors and the reviewers. Any product that may be evaluated in this article, or claim that may be made by its manufacturer, is not guaranteed or endorsed by the publisher.
